# Anti-Inflammatory Potential of Ethanolic Leaf Extract of *Eupatorium adenophorum* Spreng. Through Alteration in Production of TNF-**α**, ROS and Expression of Certain Genes

**DOI:** 10.1093/ecam/neq033

**Published:** 2011-06-16

**Authors:** Ashim K. Chakravarty, Tamal Mazumder, Shankar N. Chatterjee

**Affiliations:** ^1^Immunology and Cell Biology Laboratory, Department of Zoology, School of life Sciences, University of North Bengal, Siliguri 734013, India; ^2^Former Joint Director, SeriBiotech, Ministry of Textiles, Government of India, Kolkata, India

## Abstract

Search for a novel anti-inflammatory agent from a herbal source, such as *Eupatorium adenophorum* Spreng., a plant from the Eastern Himalayas, is of prime interest in the present investigation. Inflammation causes tissue destruction and development of diseases such as asthma, rheumatoid arthritis, and so forth. The ethanolic leaf extract of *E. adenophorum* (EEA) was administered intravenously and in other cases topically at the site of delayed type hypersensitivity (DTH) reaction in mouse foot paw induced with dinitrofluorobenzene. EEA can effectively inhibit DTH reaction and bring back normalcy to the paw much earlier than the controls. Efficacy of EEA on regulatory mechanisms for inflammation has also been considered. Intravenous administration of EEA increased the number of CD4^+^ T cells in spleen and tumor necrosis factor (TNF)-**α** in serum of DTH mice. Initially it was difficult to reconcile with the anti-inflammatory role of EEA and simultaneous induction of TNF-**α**, an established pro-inflammatory cytokine. EEA induces higher expression of *TNF-**α*** gene and amount of the cytokine in serum. We discussed the other role of TNF-**α**, its involvement in repairing tissue damage incurred in course of inflammatory reaction. EEA also induces TGF-**β** encoding a cytokine involved in tissue repair mechanism. EEA inhibits expression of another pro-inflammatory cytokine gene *IL-1**β*** and downregulates cycloxygenase 2 (*COX2*) gene responsible for metabolism of inflammatory mediators like prostaglandins. Furthermore, anti-inflammatory role of EEA is also revealed through its inhibition of hydroxyl radical generation. Notably EEA does not necessarily affect the expression of other inflammation-related genes such as *IL-6, IL-10* and *IKK*. The present study reports and analyzes for the first time the anti-inflammatory property of the leaf extract of *E. adenophorum*.

## 1. Introduction

Inflammation is the body's way of dealing with infections, maintaining a subtle balance between the beneficial effects of inflammation cascades to restrict the infection and potential for long-term tissue destruction [1–3]. If not controlled, inflammation can lead to development of diseases such as chronic asthma, rheumatoid arthritis, multiple sclerosis, inflammatory bowel disease, and so forth, [4–9]. Till date a very few anti-inflammatory drugs from herbal origin have been found, and a number of plants from ethno-medicinal databases are under laboratory investigation across the world [10].

**Figure 1:**
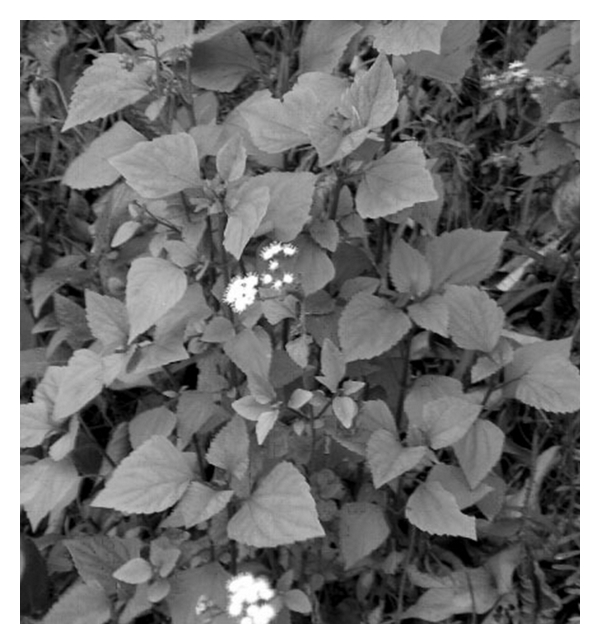
Photograph of *E. adenophorum* Spreng. in its natural habitat.

The plant *Eupatorium adenophorum* Spreng. (Figure 1) belongs to the family Asteraceae (Compositae) [11]. A number of plants of this family are commonly used in folklore medicine in different parts of the world [12–19]. In Kurseong and Darjeeling hill region of the Eastern Himalayas, local people use leaves of *E. adenophorum* Spreng., growing at an altitude of 800–2050 m, for remedial purposes against oral and skin sores. These observations suggested a probable anti-inflammatory and immunomodulatory activity of the plant's leaf extract. Earlier Mandal et al. [20] reported analgesic property of methanolic extract of the leaves. The present investigation intends to explore the anti-inflammatory property of ethanolic leaf extract of *E. adenophorum* Spreng. (EEA) in delayed type hypersensitivity (DTH) induced by 2,4-dinitrofluorobenzene (DNFB) in mouse model.

DTH reaction is initiated by pre-sensitized CD4^+^ T_DTH_ cells [21] and then other inflammatory cells and cytokines are involved at the site of reaction. The number of CD4^+^ T cells in course of DTH reaction and treatment with EEA has been enumerated to understand the effect of EEA on these cells. TNF-*α* is the most important cytokine that plays a major role in all the inflammation reactions. Serum TNF-*α* of DTH mice has also been investigated in the present study in course of inflammation.

Many reports reveal that reactive oxygen species (ROS) play an important role in developing various pathophysiological conditions including inflammation [22–25] and potent anti-inflammatory agents can scavenge the free radicals to quench the biochemical fire [26–28]. The hydroxyl radical (OH^−^) especially plays a crucial role in developing inflammation. Scavenging of hydroxyl radical (OH^−^) by EEA has been studied.

Besides TNF-*α*, many other cytokines play a key role in orchestrating immune responses in inflammation [29–36]. Here, expression of some cytokine genes such as TNF-*α*, IL-1*β*, IL-6, IL-10 and TGF-*β* in splenic T cells of DTH mice has been studied at transcription level with and without intravenous (i.v.) application of the plant extract. Expression of inhibitory kappa kinase (*IKK*) gene has also been judged. The enzyme degrades I*κ*B subunit to release active NF-*κ*B [37, 38], that is, involved in activating inflammatory responses as shown by Tak and Firestein [39] and Yamamoto and Gaynor [40].

The expression of COX1 and COX2 genes encoding two isozymes of cycloxygenase has been taken into account here. Cycloxygenase is known to play a significant role in induction of inflammation by producing inflammatory mediators like prostaglandins and leukotrienes from arachidonic acid [41–47].

## 2. Methods

### 2.1. Animal

Inbred adult Swiss Albino mice of both sexes, 12–16 weeks old have been used. They are maintained in our animal house with food and water *ad libitum*. Animals of approximately equal age and weight were used for experimental and control groups in the experiment. The experimental protocols used in the study have been approved by the Animal Ethical Committee (Regn No. 840/ac/04/CPCSEA).

### 2.2. Extract Preparation

Extract was prepared from fresh leaves of the plant *E. adenophorum* using the protocol outlined earlier [48–51]. Leaves were collected from their natural habitat at about 1400 m high slope of the Eastern Himalayas, mainly around Kurseong hill. The scientific identification of the plant has been checked by Professor A. P. Das, Plant Taxonomy Lab., Department of Botany, University of North Bengal. The leaves were cleaned thoroughly with water and allowed to air dry. In total, 10 g of leaves were crushed to a paste with a mortar and pestle. An amount of 10 mL of absolute alcohol (ethanol) was added to the paste and kept in a refrigerator overnight for extraction. The alcoholic extract was then filtered first through Whatmann filter paper and the filtrate was refiltered again through cellulose acetate filter paper (0.2 *μ*m porosity, Sartorius) for sterilization and finally stored in airtight sterilized vial at 4°C. The extract was used as such for different experiments.

### 2.3. Experimental and Control Sets and Statistical Analysis

In all the experiments, the effect of EEA was compared with two sets of control, one with equivalent amount of ethanol present in EEA and the other without any treatment. Each experiment was done on the basis of triplicate readings and such an experiment was repeated thrice or more. Results are expressed as Mean ± SD of at least nine observations. Statistical significance was analyzed using one-way ANOVA software package.

### 2.4. Induction of Delayed Type Hypersensitivity Reaction with 2,4-DNFB and Application of EEA

Delayed type hypersensitive (DTH) reaction was induced in mouse foot paw by subcutaneous application of 2,4-DNFB [48, 52]. Primary sensitization was carried out by applying 0.0001% DNFB subcutaneously in the right foot pad. After 8 days, mice were resensitized with 0.000001% DNFB on the left foot pad. Two different volumes of percentage solutions of DNFB, 25 or 50 *μ*L for both sensitization and resensitization, were used in separate experimental setups. The day of resensitization was considered as “0" day for enumeration of DTH reaction. The size of the left paw before resensitization was considered as normal size for the paw. The degree of inflammatory swelling set in the resensitized left paw was measured by a slide caliper. The effect of EEA on DTH reaction set in by two different doses of DNFB was judged after topical or i.v. application of the extract. For topical application, 5 *μ*L of EEA was applied on the resensitized paw per day from first day of resensitization. For i.v. administration, 25 *μ*L of EEA was used 1 h prior to resensitization. The percentage of inhibition of inflammation by EEA in reference to the ethanol control has been calculated by using the following formula:

(1) $$\begin{array}{cc} & \text{Inhibition}\;\text{ of}\;\text{ DTH }(\%)\\ & =\frac{\text{Untreated }\;\text{DTH}\;\text{ paw }\;\text{size}-\text{Experimental}\;\text{ paw }\;\text{size}}{\text{Untreated }\;\text{ DTH}\;\text{ paw size}}\times 100. \end{array}$$

### 2.5. Isolation of CD4^+^ T Cells Through Magnetic Assorted Cell Sorter (MACS)

The splenic lymphocytes were obtained from untreated DTH mice and mice intravenously injected with EEA and ethanol after 24, 48 and 72 h of resensitization following the protocol of Chakravarty and Maitra [53]. Erythrocytes in the spleen cell suspension were lysed by exposure to Tris-buffered ammonium chloride (0.83%, pH 7.2). For depletion of adherent cells, the suspension was incubated in a plastic petri dish at 37°C in humidified atmosphere for 30 min. Non-adherent lymphocyte population was collected and centrifuged and finally resuspended at a concentration of 10^7^ cells in 80 *μ*L. To the aliquot of 80 *μ*L cell suspension, 20 *μ*L of CD4^+^ (L3TH) microbeads (130-049-201, Miltenyi Biotech, Germany) [54–56] with a magnetic probe was added in the test tube. The tubes were refrigerated at 4–6°C for attachment of the bead to the CD4^+^ cells for 15 min. The mixture of cells and magnetic beads is then poured into the magnetic separation (MS) column fitted in the slot of the magnet of MACS. The unlabeled cells passed through the column and were collected in a tube. The MS column was removed from the separator and placed in a fresh collection tube. Of PBS, 1 ml was pipetted onto the MS column and labeled CD4^+^ cells were flushed out from the column by firmly pushing the designated plunger into the column. The magnetic labeled CD4^+^ cells were then counted in a hemocytometer.

### 2.6. Estimation of Serum TNF-*α*

The quantitation of serum TNF-*α* of DTH bearing mice treated (i.v.) with EEA and control mice was performed by solid phase sandwich enzyme-linked immunosorbent assay (ELISA) kit (Pharmingen, USA) following the protocol outlined by Paul et al. [57–59].

### 2.7. Hydroxyl Radical Scavenging Assay

The hydroxyl radical (OH^−^) was generated from Fe^2+^-ascorbate-EDTA-H_2_O_2_ system (Fentons' reaction). Assay reaction mixture was prepared by mixing a 20 mM phosphate buffer, 2 mM FeCl_3_, 1 mM EDTA, 2.8 mM 2-deoxy d-ribose, 1 mM H_2_O_2_ and 1 mM l-ascorbic acid. OH^−^ reacts with the deoxy d-ribose and a series of reaction follows to form malonaldehyde (MDA) [60]. An aliquot of 1 ml of the reaction mixture was added in each tube of experimental, alcohol control and normal control sets and incubated at 37°C for 1 h. Two different doses of EEA, 10 and 25 *μ*L, were tested for scavenging hydroxyl radical. In alcohol and normal control, same volume of ethanol and water was added respectively. After incubation, 2 mL of TBA–TCA reagent was added in each tube and boiled for 15 min for generation of MDA. MDA generated was measured at 552 nm in a spectrophotometer. The effect of both EEA and alcohol on generation of hydroxyl radical has been expressed as percentage of inhibition in MDA generation over normal control sets. The formula used is given below:

(2) $$\begin{array}{c} \text{Percentage}\;\text{ of}\;\text{ inhibition} \end{array}=\left[100-\frac{\text{MDA generated in}\;\text{ experimental}/\text{alcohol control tubes}}{\text{MDA generated in normal control tubes}}\right]\%.$$

### 2.8. Gene Expression Analysis

Expression of the genes of interest in this study has been carried out using single-cell RNA phenotyping procedure as outlined by Rappolee et al. [47, 61, 62].

### 2.9. RNA Isolation

RNA was isolated from splenic T cells of nine mice in each group using RNeasy Mini kit (74104, Qiagen, Valencia, CA, USA), as per manufacturer's protocol. Briefly, 6 × 10^6^ T cells were homogenized with a 300 *μ*L RLT buffer and passing them through a 2 mL syringe fitted with a 27-gauge needle. Of 70% ethanol, 300 *μ*L was added to the homogenate and collected in a spin column fitted upon a collection tube. The spin columns and collection tubes were supplied by the manufacturer. After a brief centrifugation for 15 s at 10 000 rpm, the fluid was passed into the collection tube that was then decanted and reattached to the spin column. With addition of 500 *μ*L of buffer RW1 into the spin column centrifugation was made again for 15 s at 10 000 rpm. Following decantation of collection tube, 500 *μ*L of buffer RPE was added to the spin column and centrifuged similarly, and the step was repeated one more time. Finally, the spin column was fitted upon a fresh collection tube and washed twice with 15 *μ*L of DEPC-treated water to come up with a total of 30 *μ*L volume containing the RNA sample.

The concentration of RNA was measured spectrophotometrically at 400× dilution with Shimadzu UV-160, Japan. The extracted RNA was used for cDNA synthesis.

### 2.10. cDNA Synthesis

The isolated RNA was used for First strand cDNA synthesis utilizing the RevertAid First strand cDNA synthesis kit # K1621 from Fermentas and the manufacturer's protocol was followed. For synthesis of first strand cDNA, the primer used for PCR amplification was oligo(dT^13^) synthesized by GMBH. cDNA constructed was stored at −20°C for further use.

### 2.11. Primer Utilized and Amplification Schedule

Primers were designed from various geneBank accession retrieved from PUBMED Data Bank as listed in Table 1, using the primer program available on the internet. The designed primers were synthesized by GMBH, Germany. Details of the primers are given in Table 1.

PCR was performed using a thermocycler (PeqLab, Germany) for 35 cycles in a 30 *μ*L reaction mixture containing Taq DNA polymerase buffer, all four dNTPs, oligonucleotide primers, Taq DNA polymerase and cDNA products. After amplification, PCR products were analyzed on a 0.8% (w/v) agarose gel. The band density was quantified on the basis of the known concentration of lambda DNA (30 ng) through ImageAide, Spectronics Corporation, NY.

## 3. Results

### 3.1. Effect of EEA on Delayed Type Hypersensitive Reaction

#### 3.1.1. With Topical Application

**Figure 2:**
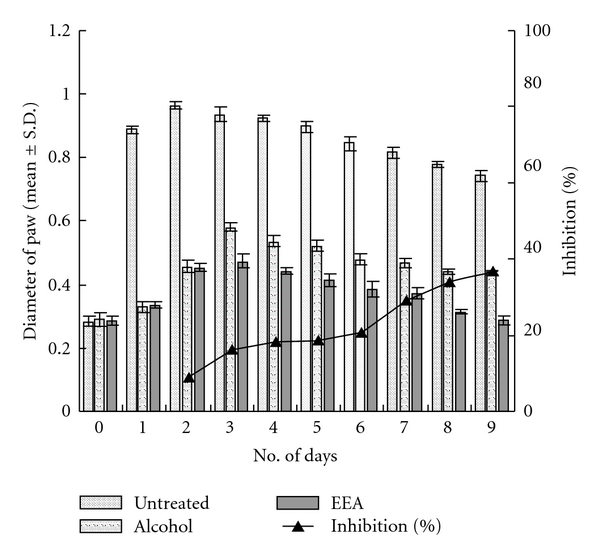
Inhibitory effect of topical application of EEA on delayed type hypersensitive (DTH) reaction induced with 25 *μ*L 2,4-DNFB (significance at *P < .01).

Figure 2 represents the data of delayed type hypersensitivity reaction induced with 25 *μ*L DNFB and treatment with ethanolic leaf extract of *E. adenophorum* (EEA) (experimental) and ethanol in control. Swelling of resensitized paw in both control and experimental mice was maximum on third day; with topical application of EEA, the maximum swelling was in the range of 0.4733 ± 0.0227 cm, and in ethanol-treated control mice the maximum value was 0.5667 ± 0.173 cm (Figure 2). The paw size in EEA-treated mice gets back to normal range by the ninth day of resensitization. Inhibition with EEA is a continuous process throughout the experiment. Figure 2 also represents the percentages of inhibition on different days.

**Figure 3:**
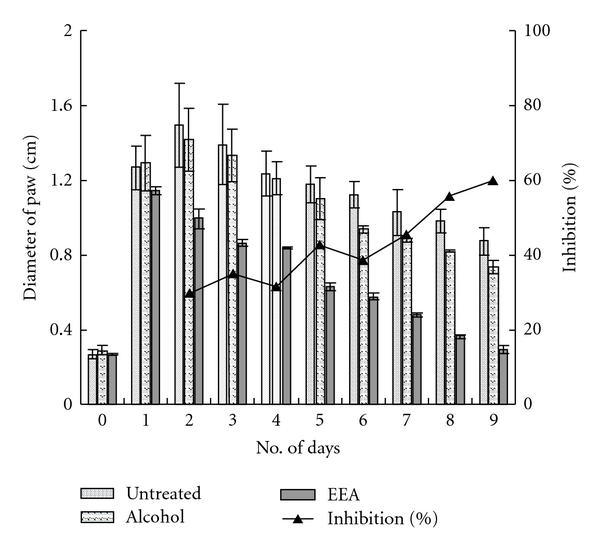
Indurations of DTH swelling, induced with 50 *μ*L DNFB, and inhibition after topical application of EEA and alcohol (significance at *P < .01).

**Figure 4:**
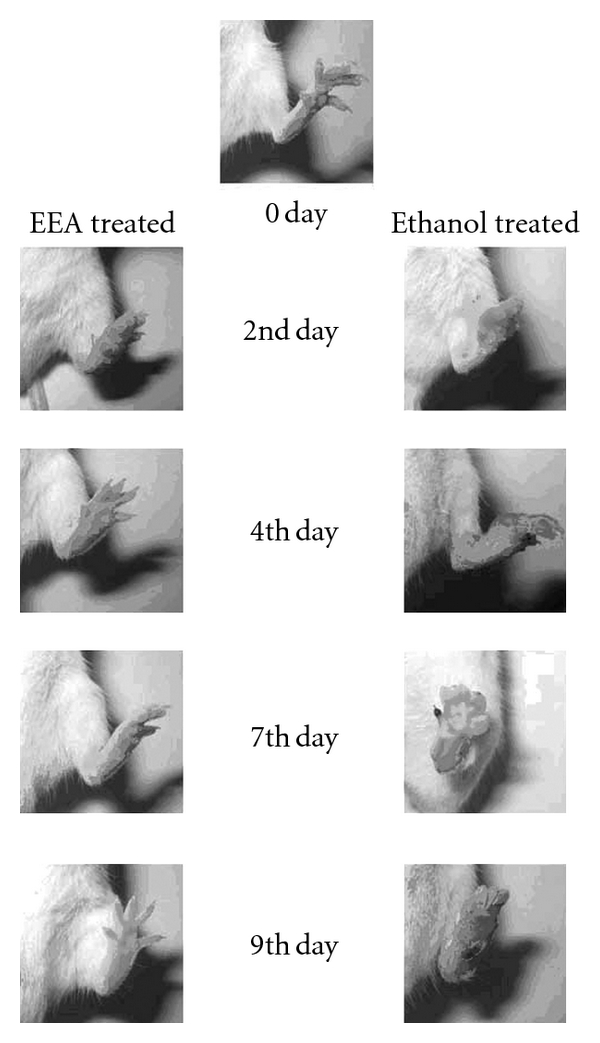
Photographs showing effect of topical application of EEA and alcohol on resensitized paw of DTH mice, induced by 50 *μ*L DNFB, during 9-day period of the study.

A higher dose (50 *μ*L) of DNFB causes the swelling to be more and the peak on the second day; however, recovery with EEA treatment is on the ninth day (Figure 3). In fact EEA inhibited the swelling by 60% on the ninth day.  Figure 4 is the photographic representation of DTH reaction induced with 50 *μ*L DNFB and inhibition with EEA treatment.

#### 3.1.2. With i.v. Application

**Figure 5:**
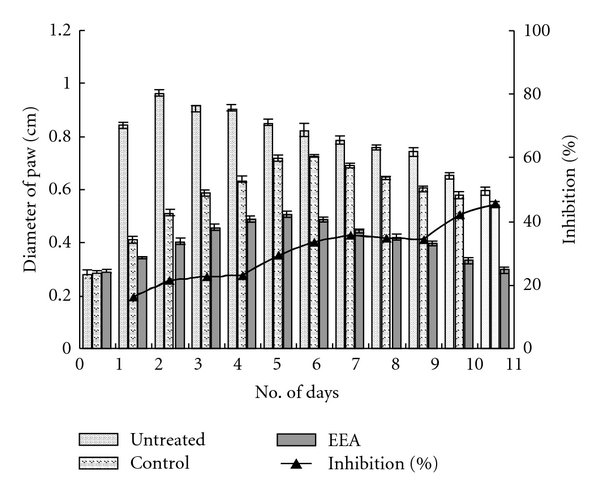
Changes in paw size of DTH mice, induced with 25 *μ*L DNFB, and after i.v. application of EEA and alcohol (significance of inhibition by EEA at *P < .01 and **P < .05).

When EEA was administered intravenously in DTH bearing mice, inflammatory swelling of the resensitized paw persisted longer than in cases of topical application of EEA. The dose of 25 *μ*L DNFB could induce maximum swelling of 0.963 ± 0.012 cm on the second day in untreated mice. Intravenous application of EEA caused the DTH reaction to slow down. In alcohol group the swelling was maximum, 0.7367 ± 0.0045 cm, on fifth day (also on sixth). EEA treatment restricted the swelling to 0.5123 ± 0.0112 cm on fifth day and brought back normalcy (0.2967 ± 0.0103 cm) by 11th day; the inhibition over the ethanol control was found to be 45.39% (Figure 5).

**Figure 6:**
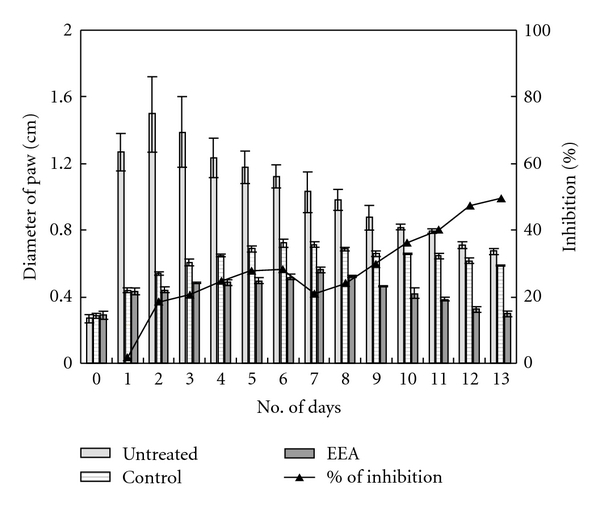
Changes in paw size of DTH mice, induced with 50 *μ*L DNFB, and after i.v. application of EEA and alcohol (significance of inhibition by EEA at *P < .01).

The degree of swelling was more at DTH site with higher dose (50 *μ*L) of DNFB (Figure 6.) inhibited the reaction more effectively than alcohol alone (control) and allowed regaining normalcy by 13 days (Figure 6).

### 3.2. Estimation of the Number of CD4^+^ T Cells in DTH Mice

**Figure 7:**
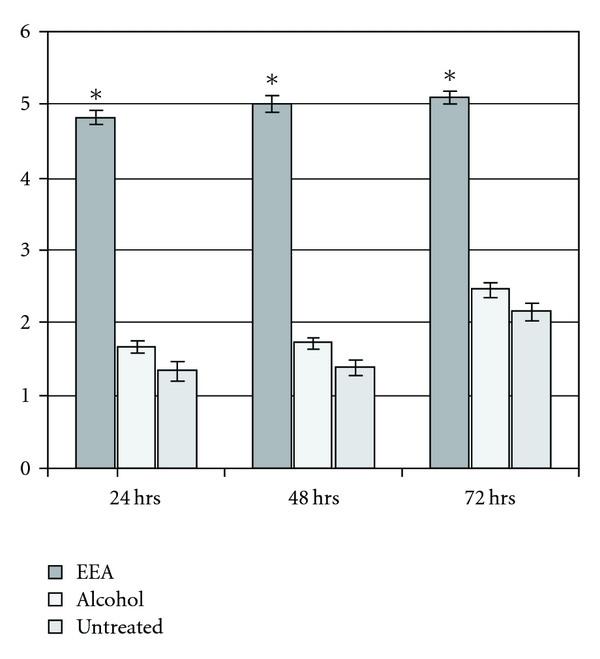
Effect of intravenously administered EEA and alcohol on count of CD4^+^ T cells from spleen of mice after 24, 48 and 72 h of induction of DTH with 25 *μ*L DNFB (significance of results with EEA over control at *P < .01).

CD4^+^ T cells from splenic lymphocyte population were isolated by labeling with microbeads containing a paramagnetic probe and passing through MACS. The number of CD4^+^ T lymphocytes in the spleen of DTH mice treated with EEA was significantly more than the controls, about two and a half times at 24 and 48 h and two times at 72 h (Figure 7). A slight increase in the cell number in the alcohol control group was noted (Figure 7).

### 3.3. Serum TNF-*α* of DTH-Bearing Mice and Upon EEA Treatment

Serum TNF-*α* in three groups of mice bearing DTH reaction—untreated, alcohol and EEA treated (i.v.)—was measured by a solid phase sandwich enzyme-linked immunosorbent assay (ELISA).

**Figure 8:**
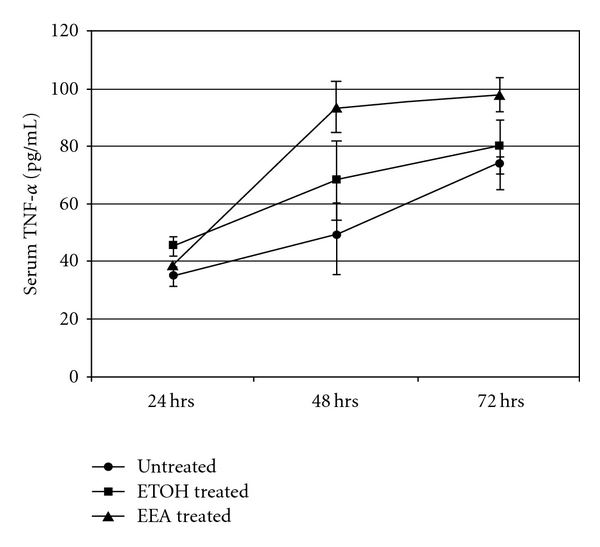
Level of serum TNF-*α* from DTH mice (induced with 25 *μ*L DNFB) at three different hours in presence and absence of EEA (significance compared to controls at *P < .01).

There was no appreciable difference in the level of serum TNF-*α* in the three groups of mice at 24 h (Figure 8). The TNF-*α* level in DTH mice treated with EEA (i.v.) was more than that in other groups by 48 h (93.23 pg mL^−1^) and the level was maintained upto 72 h (Figure 8).

### 3.4. Generation of Hydroxyl Radical and Effect of EEA

**Figure 9:**
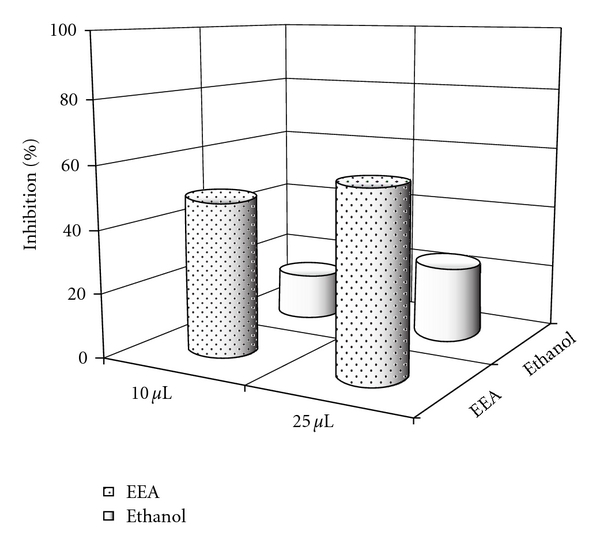
The effect of two different doses of EEA on inhibition of generation of hydroxyl radical (OH^•^).

The hydroxyl radical (OH^•^) is potentially harmful for the cellular macromolecules and is implicated in pathophysiology of inflammation. EEA inhibits hydroxyl radical generation up to 57.98% at a dose of 25 *μ*L. The same dose of ethanol (control) showed 23.62% inhibition. At a lower dose of 10 *μ*L, the degree of inhibition was about 49.25% in experimental ones and 14.88% in case of alcohol control (Figure 9).

### 3.5. Expression of Certain Genes in Splenic T Cells of DTH Mice with and without EEA Treatment

**Figure 10:**
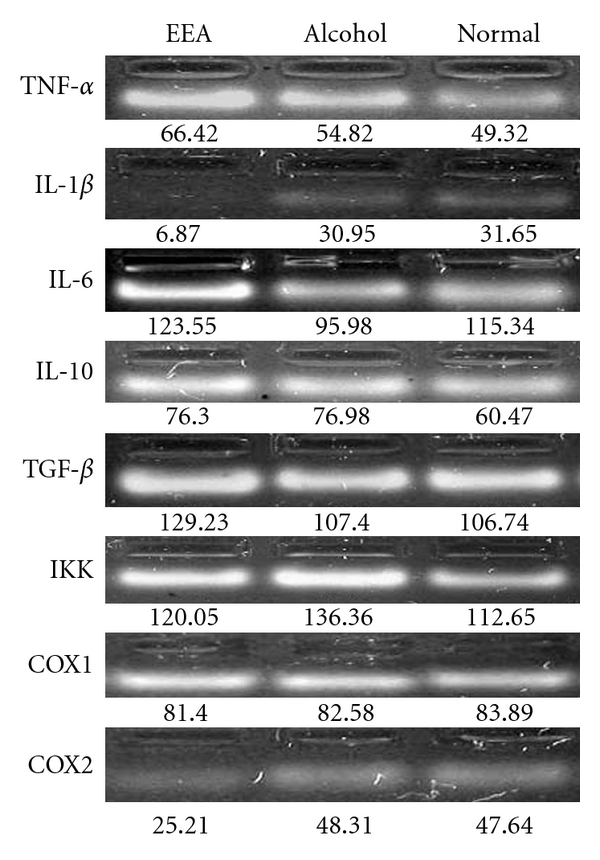
Expression of certain inflammation-related genes in T cells obtained from DTH mice, and with and without EEA treatment, containing nine mice in each group. Agarose gel electrophoresis of cDNA PCR products amplified with different gene-specific primers.

**Figure 11:**
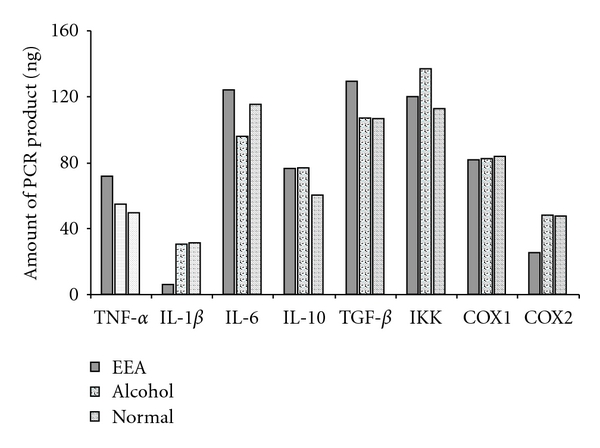
Graphical presentation of quantification of the DNA bands presented in Figure 10 showing expression of the inflammation-related genes in T cells of DTH mice with and without EEA treatment, containing nine mice in each group. The DNA bands were quantified against 30 ng of lambda DNA through ImageAide, Spectronics Corporation, NY.

The T cells actively participate in the progression of DTH reaction. Up- or downregulation of certain genes in the cells must be related to the production of pro- and anti-inflammatory cytokines, transcription factors and mediators. The level of expression of some of these genes at transcription level in splenic T cells of DTH mice untreated and treated with EEA was judged, by quantitating the cDNA PCR product amplified with specific primers. The quantitation was done against 30 ng of lambda DNA as a standard using ImageAide, Spectronics Corporation, NY. The data are presented in Figures 10 and 11.

EEA caused an increment in the expression of *TNF-*α**, a pro-inflammatory cytokine (Figures 10 and 11). At the same time, EEA inhibited expression of pro-inflammatory cytokine *IL-1*β** and showed no effect on *IL-6*, another pro-inflammatory cytokine.

EEA did not influence the expression of *IL-10*, an anti-inflammatory cytokine beyond alcohol control. The expression of *TGF-*β** encoding a cytokine involved in regeneration was induced by EEA beyond the level in the controls.

EEA apparently did not influence expression of *IKK* and *COX1* genes but downregulated the expression of *COX2* gene.

## 4. Discussion

The ethanolic leaf extract of *E. adenophorum* Spreng. (EEA) could effectively suppress the inflammatory reaction set in foot paw by injecting two different doses of 2,4-DNFB (Figure 4). The topical application of EEA was more effective in inhibition of the swelling of foot paw and gaining normalcy faster than its i.v. application (Figures 2, 3, 5, and 6).

EEA treatment caused significant increase in the number of the CD4^+^ T cell population in DTH mice (Figure 7). These cells are known to play a central role in inflammatory reactions by secreting all different kinds of cytokines that regulate participation of other kinds of cells [21, 63, 64].

TNF-*α* is a major cytokine involved in DTH reaction. EEA induced higher level of serum TNF-*α*, surpassing the level in mice undergoing DTH reaction or in mice treated with alcohol (Figure 8). It is worthwhile to note EEA capable of inhibiting DTH reaction is inducing a pro-inflammatory cytokine like TNF-*α*. Banno et al. [65] showed that TNF-*α* promotes tissue repair of damage skin by inducing basement membrane components and collagen degrading proteases to participate actively in reconstruction of extracellular matrix. Kuwano and his coworkers [66] found that TNF-*α* can also induce growth-promoting event like angiogenesis by increasing mRNA level of IL-8, vascular endothelial growth factor and fibroblast growth factors in endothelial cells. We think of similar participation of TNF-*α* in tissue repair and regeneration to bring back normalcy aftermath of DTH reaction. Thus, TNF-*α* plays a double role in DTH reaction—pro-inflammatory cum restoring agent.

The ability of EEA for induction of *TNF-*α** gene was studied next. Indeed, it induced higher level of expression of the gene (Figures 10 and 11). EEA does not necessarily affect the expression of gene for other pro-inflammatory cytokines such as *IL-1*β** and IL-6 in a similar fashion. EEA inhibited *IL-1*β** expression and did not influence the expression of *IL-6* gene (Figures 10 and 11). It seems that IL-1*β* without any known function in repair mechanism manifests more inhibitory effect of EEA on inflammation. Stimmeder and his co-workers [67] observed that lornoxicam and other non-steroidal anti-inflammatory drugs inhibit *IL-1*β** expression as well as inflammation. Kohli et al. [68] reported that curcumin, the active component in the rhizome of *Curcuma longa* Linn., demonstrates its anti-inflammatory activity by inhibiting production of IL-1*β* in lung inflammatory cells.

An anti-inflammatory agent does not necessarily always regulate all the anti-inflammatory cytokine genes as we find that in the present study EEA does not influence the anti-inflammatory cytokine gene *IL-10* (Figures 10 and 11).

EEA upregulates expression of *TGF-*β** (Figures 10 and 11). TGF-*β* performs as a growth factor in all different kinds of events of collagen production and extracellular matrix reorganization as shown by Barcellos–Hoff [69]. This cytokine might function here to restore normalcy along with TNF-*α* in repair mode as discussed earlier. Simultaneous upregulation of these two genes have also been observed by Chao et al. [70] in microglial cell culture. Sullivan et al. [71] also reported similar trend in expression of these two cytokines in interstitial pulmonary fibrosis-affected lung fibroblasts.

Tak and Firestein [39] and Yamamoto and Gaynor [40] elucidated involvement of NF-*κ*B pathway for induction of inflammation. Activation of NF-*κ*B is mediated by the action of Inhibitory kappa kinase (IKK) degrading inhibitory I*κ*B subunit. Thus, measuring the expression of *IKK*, one can derive the involvement of NF-*κ*B pathway in a reaction. EEA could not induce the expression of *IKK* gene beyond the controls (Figures 10 and 11) indicating non-involvement of NF-*κ*B activation pathway for DTH reaction induced with DNFB.

COX1 and COX2 gene products are two isoforms of the cycloxygenase enzyme that metabolizes arachidonic acid into the inflammatory mediators like prostaglandins and leukotrienes [41–47]. Notably EEA only influences expression of *COX2* gene by way of inhibition (Figures 10 and 11). This may be another way of execution of anti-inflammatory activity by EEA. Salvioli et al. [72] reviewed curcumin, a potent anti-inflammatory agent, also inhibits COX2 in abetting inflammation.

It is known that reactive oxygen species play an important role in development of inflammation [22–25]. In the present study, the ability of EEA in quenching the generation of hydroxyl radical has been tested and found to be effective (Figure 9).

**Figure 12:**
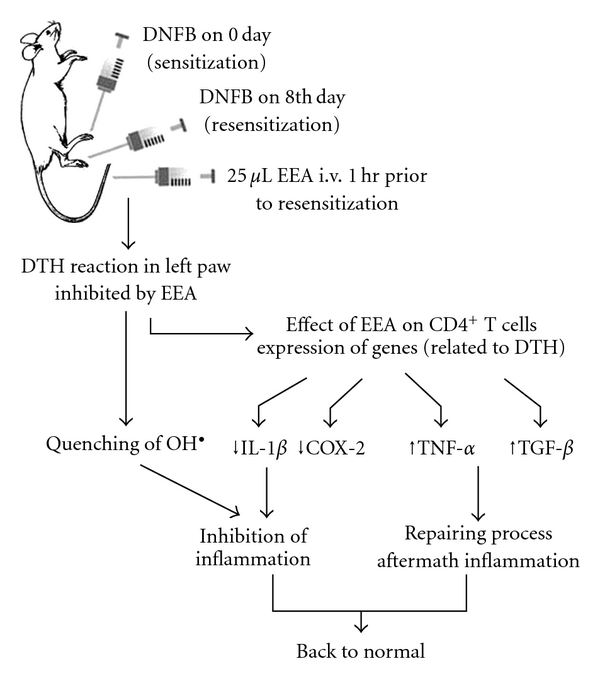
Effect of EEA on inhibition of DTH reaction and repair mechanism presented schematically.

In summary, ethanolic leaf extract of *E. adenophorum* (EEA) exerts anti-inflammatory activity (Figure 12), likely through inhibition of *IL-1*β**, *COX2* genes and quenching ROS like hydroxyl radical. Simultaneously EEA induces production of TNF-*α*, a pro-inflammatory cytokine. This paradox can only be resolved in the light of participation of TNF-*α* in tissue repair in the aftermath of inflammation. Interestingly, the expression of *TGF-*β** gene encoding the cytokine responsible for growth and repair mechanism is also inducible by EEA. *E. adenophorum* as a source of anti-inflammatory substance seems worthy to report. The active compound from the extract is yet to be identified. Isolation of that compound will allow understanding molecular mechanism of the activity of the substance. So far, Zhang et al. [73] reported presence of a few flavonones and sesquiterpene lactones in *E. adenophorum*.

The present investigation also intends that any herbal agent having anti-inflammatory property might be screened faster by estimating its ability to induce or inhibit the genes encoding substances participating in inflammation.

## Funding

University Grants Commission (UGC), India.

## Figures and Tables

**Table 1:** Primers.

Primers	Accession number	Sense (5′–3′)	Antisense (3′–5′)	*T*_m_ (°C)
IKK	NM_010546	CCAGACTCCAAGGTGGTGTT	TGCAGATCACAGGCAGAAAC	60.0
TNF-*α*	NM_013693	TGGCACAGCCAAG	GGGACCCCTGCTC	52.36
TGF-*β*	NM_011577	TTGCTTCAGCTCCACAGAGA	TGGTTGTAGAGGGCAAGGAC	59.99
IL-1*β*	NM_008361	GTGGCAGCTACCTGTGTCTT	GGAGCCTGTAGTGCAGTTGT	57.96
IL-6	NM_031168	GGGAAATCGTGGA	AGGTTTGCCGAGT	43.9
IL-10	NM_010548	CCAAGCCTTATCGGAAATGA	TTTTCACAGGGGAGAAATCG	60.035
COX1	BC023322	AGAAACTGGTCTGCCTCA	AACCCACATCAAGGACTG	54.02
COX2	NM_011198	AGCACCATTCTCCTTGAA	GTAGGCTGTGGATCTTGC	54.0
PKC-*θ*	NM_008859	AAGTGAGAAACCCCGGCTAT	AGGCAAATCCCTTCCAGTCT	60.01
Perforin	NM_011073	ACCCTGAATGGGCTCACA	GCAGCAGTCCTGGTTGGT	57.0
